# 达沙替尼治疗Ph^+^急性淋巴细胞白血病致视神经炎1例

**DOI:** 10.3760/cma.j.issn.0253-2727.2023.07.019

**Published:** 2023-07

**Authors:** 磊 傅, 磊 沈, 建军 卞, 亮 李, 玉璇 苏, 金曼 左, 美丽 孟, 尧 陆, 书亚 葛, 杜传 王

**Affiliations:** 蚌埠医学院第二附属医院血液内科，蚌埠 233020 Department of Hematology, the Second Affiliated Hospital of Bengbu Medical College, Bengbu 233020, China

患者，男，51岁。2020年1月因“发热1周”就诊于当地医院。血常规：WBC 95.43×10^9^/L，HGB 130 g/L，PLT 103×10^9^/L，遂转至我院。查体：颈部双侧及腋窝可触及肿大淋巴结，质韧，无压痛，胸骨轻压痛，肝脾肋缘下未触及。骨髓细胞形态学及免疫分型提示为急性B淋巴细胞白血病（B-ALL）。融合基因检测：BCR-ABL融合基因（P190型）阳性；染色体核型：45，XY，−7，der（9）t（7；9）（p13；p22）t（9；22）（q34；q11.2），der（22）t（9；22）（q34；q11.2）[20]。诊断为Ph^+^ ALL。2020年1月17日予伊马替尼400 mg/d治疗，并VDCP方案（长春地辛+环磷酰胺+伊达比星+泼尼松）诱导化疗1个疗程，后复查骨髓，疗效评价为完全缓解（CR）。给予Hyper-CVAD、大剂量甲氨蝶呤（HD-MTX）/阿糖胞苷（Ara-C）方案交替巩固化疗共8个疗程，后给予VP方案（长春地辛+泼尼松）每月1次维持化疗共2年，期间一直予伊马替尼治疗，并多次鞘内注射（鞘注）Ara-C、MTX、地塞米松预防中枢神经系统白血病，疗效评价持续CR且BCR-ABL融合基因阴性。

2022年3月入院复查，骨髓细胞形态学及免疫分型提示ALL复发，BCR-ABL融合基因（P190型）阳性，BCR-ABL/ABL：88.31％。染色体核型：45，XY，−7，der（9）（q22q34），der（9）t（7；9）（p13；p22）t（9；22）（q34；q11.2），der（22）t（9；22）（q34；q11.2）[20]。ABL激酶区未见突变。给予达沙替尼100 mg/d联合VDCP方案诱导化疗1个疗程，行腰椎穿刺术未发现白血病中枢神经系统浸润，给予鞘注预防治疗，后复查骨髓再次CR。2022年4月给予hyper-CVAD+达沙替尼方案巩固化疗1个疗程，并鞘注预防治疗，后患者拒绝继续进行联合化疗，一直口服达沙替尼治疗，复查骨髓均CR中，BCR-ABL融合基因持续阴性。

2022年7月患者无明显诱因下出现视力下降，后进行性加重直至双眼视物不能，仅存少量光感。入院复查骨髓细胞形态学、免疫分型未见异常，BCR-ABL融合基因阴性，染色体核型为正常核型，提示仍CR中。腰椎穿刺术检查提示脑脊液压力正常，脑脊液常规、生化、脱落细胞学及免疫分型均未见异常。眼眶MRI平扫+增强：双侧视神经肿胀伴异常强化，考虑视神经炎。颅脑、颈椎、胸椎、腰椎MRI平扫+增强均未见明显异常。眼底镜检查未见眼底出血，但视野缺损明显，眼科、神经内科、神经外科、感染科、影像科及药剂科多学科会诊后完善相关检查：神经系统自身免疫性抗体均阴性，HIV病毒、EB病毒、巨细胞病毒、腺病毒、疱疹病毒及肝炎病毒检测均阴性，布鲁氏菌及汉赛巴尔通体均未检出，结缔组织病相关抗体均阴性，维生素B6、B1、B12和叶酸检测均正常。最终考虑为达沙替尼继发的双侧视神经炎，予停用达沙替尼。给予甲泼尼龙1 000 mg/d，静脉滴注，共5 d，后逐渐阶梯减量，后改为泼尼松口服并逐渐减量，患者双眼视力逐渐恢复，可正常视物、生活。2022年8月起给予氟马替尼600 mg/d治疗，白血病持续缓解，视神经炎症状未再发作。

